# Triple-Branch Catalytic Assembly DNAzyme Motivated DNA Tweezer for Sensitive and Reliable *mecA* Gene Detection in *Staphylococcus aureus*

**DOI:** 10.4014/jmb.2409.09008

**Published:** 2024-10-01

**Authors:** Xiaoyang Li, Meiyan Xu, Fangmin Gan, Hui Zhao

**Affiliations:** Infectious Department, People’s Hospital of Yueqing City, Yueqing City, Wenzhou City, Zhejiang Province 325600, P.R. China

**Keywords:** *mecA* gene, *Staphylococcus aureus*, DNA tweezer, catalytic hairpin assembly

## Abstract

*Staphylococcus aureus* (*S. aureus*, SA) is one of the most common bacteria in nosocomial infections. Sensitive and efficient analysis of methicillin-resistance of SA is crucial for improving the nursing performance of pneumonia. However, methicillin-resistance analysis with favorable sensitivity and specificity in an enzyme-free manner remains a huge challenge. This paper presents the development of a new fluorescent biosensor for detecting *mecA* gene using a triple-branch catalytic hairpin assembly (CHA) triggered DNAzyme switch-based DNA tweezer. The SA from the samples are immobilized on the plate’s surface using the protein A antibody. The biosensor possesses several key features. Firstly, it utilizes dual signal amplification processes, specifically the triple-branch CHA and DNAzyme controlled DNA tweezer-based signal recycling, to enable *mecA* detection on the plate. This design enhances the method’s sensitivity, resulting in a low limit of detection of 1.5 fM. Secondly, the biosensor does not rely on enzymes for *mecA* analysis, ensuring a high level of stability during target analysis. Lastly, the method demonstrates a remarkable selectivity by accurately distinguishing target sequences from non-target sequences. The proposed biosensor, which does not require enzymes and has a high level of sensitivity, offers a viable platform for the rapid and simple quantification of *mecA* in SA.

## Introduction

*Staphylococcus aureus* (*S. aureus*, SA) has emerged as an increasingly important pathogen in nosocomial infections, particularly in patients with significant health care exposure [1-3]. The methicillin-resistant *Staphylococcus aureus* (MRSA) related pneumonia is associated with poor outcomes and frequently merits empirical antibiotic consideration despite its relatively low incidence [4-6]. Due to the resistance to penicillins and other β-lactam antibiotics, MRSA is highly contagious and one of the most significant pathogens of community infection, compromising the nursing effect [7, 8]. The mechanism of antibiotics resistance in MRSA is based on incorporation with mec DNA, including *mecA*, a gene encoding penicillin-binding protein 2a (PBP 2a) [9]. Thus, the *mecA* gene can be regarded as an indicator of MRSA detection.

In the last few decades, many methods have been employed to analyze *mecA*, including massively parallel sequencing (MPS), quantitative real-time PCR (qRT-PCR) [10, 11], and microarrays [12, 13]. Each of these approaches possesses unique merits and demerits. For instance, qRT-PCR is regarded as the gold standard for *mecA* analysis due to its exceptional sensitivity and adaptability. Amplification bias and artifacts are, however, two of the most significant drawbacks of qPCR-based *mecA* quantification techniques. Moreover, due to their diminutive size, *mecA* necessitate the addition of a poly(A) tail in order to be identified using standard PCR assays [14]. Nevertheless, the efficacy of *E. coli* poly(A) polymerase is significantly influenced by the terminal nucleotide (A = G > C > U), suggesting that this method has an intrinsic predilection for specific sequences. Optimized for the identification of novel *mecA*, MPS is labor-intensive and expensive, and requires significant bioinformatics assistance. Microarray platforms, conversely, exhibit reduced specificity in comparison to qPCR or sequencing. Additional deficiencies of molecular biology-based methods that restrict their utility for routine clinical analysis are their reliance on costly, sophisticated instruments and their inability to quantify *mecA* in absolute terms. In recent years, numerous innovative techniques have been devised, representing significant advancements over conventional methods for *mecA* detection [15-18]. However, the utilization of these techniques for portable *mecA* analysis is restricted due to the requirement of multiple enzymes for signal amplification, despite the fact that they have significantly enhanced the performance of *mecA* detection.

DNA tweezers, a type of DNA nanomachine, are created by the use of Watson-Crick base pairing between several DNA sequences [19]. This allows for precise mechanical movement at the micro or nanoscale. DNA tweezers possess distinct benefits such as predictable architecture, manageable assembly, favorable compatibility with biological systems, and straightforward adaptability [20, 21]. DNA tweezers, as indicated by the nomenclature, mimic the form of conventional tweezers. These tweezers typically consist of a stiff, double-stranded DNA structure that forms the essential clamp arms. They function by regulating DNA hybridization to detect target molecules. The double arms, directed by a flexible connecting axis, can modify their position to replicate the grabbing action of surgical forceps on the target [22]. DNA tweezers have the ability to modify the opening and closing of nanostructures in response to various external stimuli, including nucleic acids, proteins, enzymes, metal ions, and pH levels.

A fluorescent biosensor for sensitive and portable *mecA* analysis is constructed in this study utilizing a triple-branch catalytic hairpin assembly (CHA) triggered DNAzyme switch-based DNA tweezer. This biosensor incorporates dual signal amplification processes, namely the target recognition-initiated triple-branch CHA process and the DNAzyme controlled DNA tweezer-based signal recycling, which endows the method a high sensitivity. Leveraging the capabilities of biosensors, this technology exhibits great promise in enabling the prompt and dependable detection *mecA*, thereby facilitating the investigation of pathological mechanisms.

## Materials and Methods

### Regents and Material

The complete list of oligonucleotides that were previously used can be found in Table S1. The sequences were synthetically produced and refined by Sangon Biotechnology Co., Ltd. (China). The protein A antibody and bovine serum albumin (BSA) were acquired from Sigma-Aldrich. The Hitachi F-7100 Fluorescence Spectrophotometer from Japan was utilized to get fluorescence spectra. The excitation and emission slit widths were both configured to 10 nm.

### Preparation of DNA Tweezers

The oligonucleotide for the DNA tweezer was dissolved in phosphate buffer saline to a concentration of 5 μM. The resulting mixture was then heated to 95°C for 5 min. Subsequently, the DNA tweezers were formed through a self-assembly process by gradually cooling it to room temperature (RT).

### Detection Performance of the Biosensor

*Fixation of SA*: 100 μl of SA with different concentrations were added to the plates and incubated at room temperature for 30 min. After the supernatant is poured, the well is cleaned three times using phosphate buffered saline-20 (PBST) as a buffer to remove any remaining unbound antigens, antibodies, or reagents. 10 μl of the PBS buffer containing BSA was added to each well.

*The mecA gene analysis*: First, 10 μl of the probe mixture containing H1, H2, and H3 probes (10 μM each) was diluted in 100 μl of serum-free DMEM, rotated for 1 second. The mixture was then added to plate containing MRSA cell extraction. The mixture was left at room temperature for 60 min to produce fluorescence signals.

## Results and Discussion

### The Working Mechanism of *mecA* Detection

**Fig. 1:**
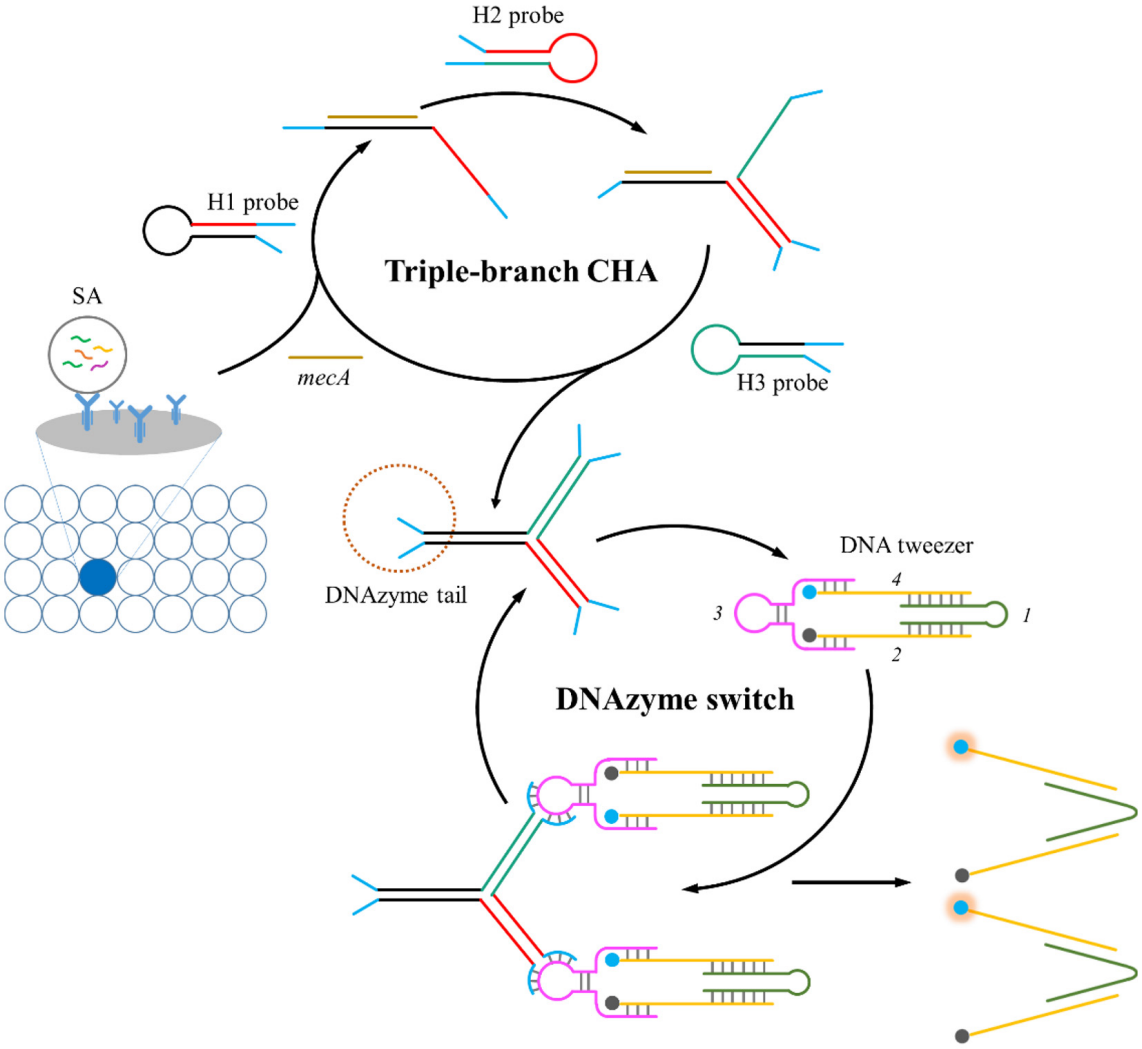
The working mechanism of the proposed approach for sensitive *mecA* detection.

Fig. 1 illustrates the concept of fluorescent biosensor for *mecA* detection. The established biosensor consists of two steps: SA fixation using protein A antibodies and sensitive detection of *mecA*. Once the SA cells are securely attached to the plate by the protein A antibody, the genomic material of the SA was extracted. During the process of signal amplification, the extracted *mecA* have the ability to selectively identify and unfold H1 probes, which in turn expose binding sites for H2 probe identification. Following the hybridization of the H2 probe with the H1 probe, a double-stranded structure containing two toeholds is created. The resulting toehold structure facilitates the opening of the H3 probe, resulting the formation of triple-branch CHA product and the release of target *mecA*. Following this, the released *mecA* mediates the signal cycle by unfolding a next H1 probe. The triple-branch CHA product with three DNAzyme tails at its terminals serves a switch to start the DNA tweezer based signaling process. Specifically, the exposed DNAzyme tails of the triple-branch CHA product can bind with the loop section of the linker (sequences 3) of the DNA tweezers and form active secondary conformation. With the assistance of metal ions, the DNAzyme tail in triple-branch CHA product generates a nicking site in the loop section of the linker, resulting in the activation of the DNA tweezers. Simultaneously, the triple-branch CHA product containing DNAzyme tails is released to bind with a next DNA tweezer, forming recycling “OPEN” of DNA tweezer, leading to the amplification of fluorescent signals.

### Fixation of SA and Feasibility of the CHA Based DNA Tweezer for Target Sensing

**Fig. 2:**
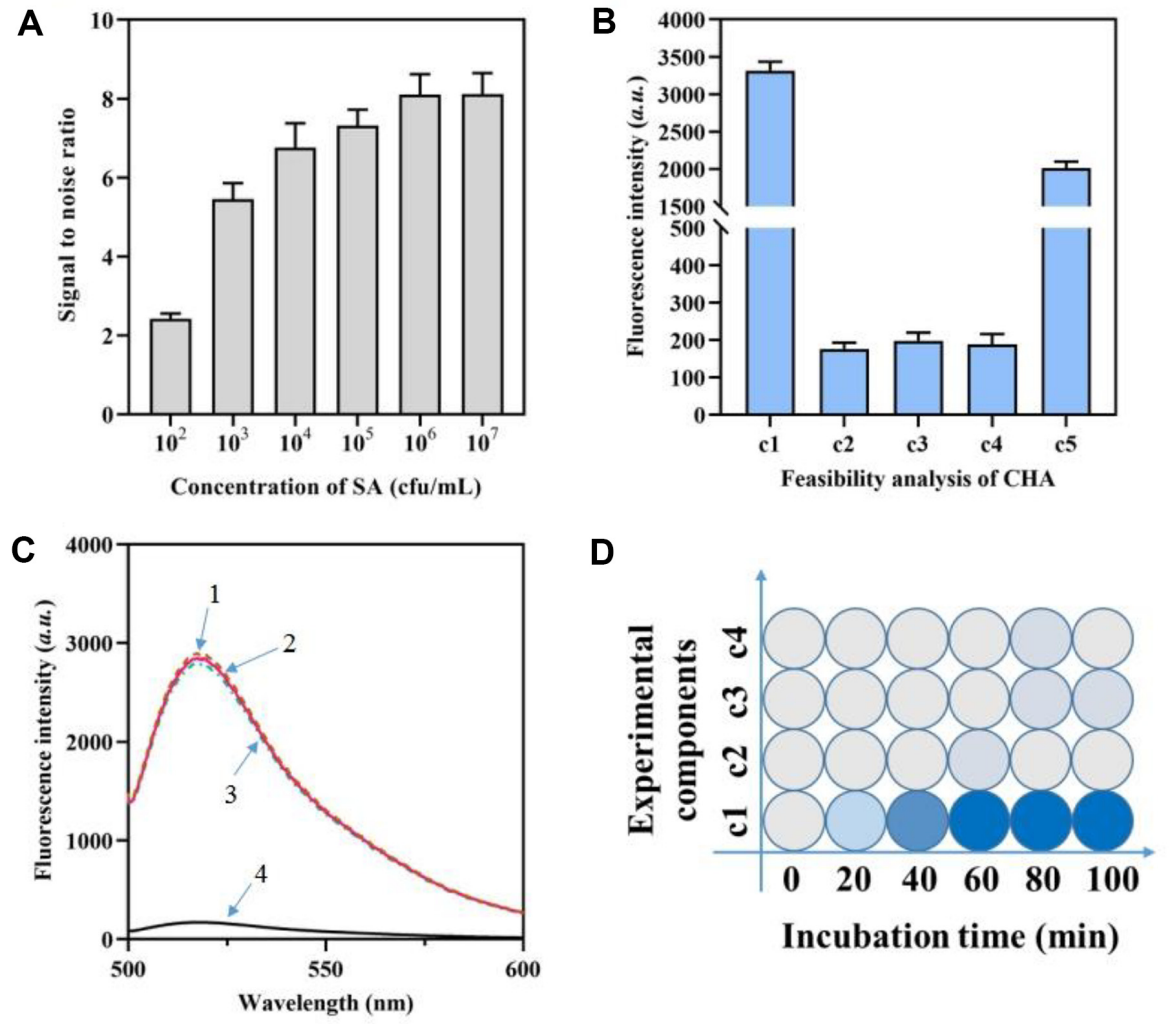
Feasibility analysis of the biosensor for *mecA* analysis. (**A**) Fluorescence ratio of the SA fixed on the surface of plate with different concentrations of SA. (**B**) Fluorescence intensity of the H3 probe during the CHA process. c1, H3 in linear state; c2, H3 (hairpin structure); c3, H3+ H1; c4, H3+ H2+ H1; c5, H3+ H2+ H1+ *mecA*. (**C**) Fluorescence spectrum of the DNA tweezer. 1, chain 1; 2, chain 1+ chain 2; 3, chain 1+ chain 2+ chain 3; 4, chain 1+ chain 2+ chain 3+ chain 4. (**D**) *In situ* *mecA* analysis with different incubation time and experimental components combinations.

Effective and precise fixation of SA is necessary before investigation of *mecA* gene. Thus, the enzyme-linked immunosorbent assay was employed to confirm the SA fixation. Fig. 2A demonstrates that as the SA concentration grew gradually, the fluorescence signal measured on the plate surface was amplified. Once the SA concentration surpassed 10^6^ CFU/ml, the fluorescence signals ceased to show any meaningful increase, suggesting that the SA fixation had achieved its maximum capacity.

Furthermore, the validity of triple-branch CHA caused by target identification has been confirmed using fluorescence tests. When the H3 sequence was in linear state, the fluorescence signal of the H3 sequence was high because FAM moiety was not quenched by the quenching moiety (c1). When the H3 sequence was assembled to hairpin structure, the recorded fluorescence signal decreased (c2). In addition, the fluorescence signal of the H3 probe remained low when H3 probe was mixed with H2 probe (c3). The mixture of H3, H2, and H1 also showed a low fluorescence intensity, indicating no non-specific hybridization between the three probes (c4). Only when target *mecA* existed, a greatly elevated fluorescence signal was recorded, indicating that the triple-branch CHA was initiated and the H3 probe was unfold (c5). This observation suggests that the H3 probe was activated and the triple-branch CHA process was successfully executed (Fig. 2B).

The construction of the DNA tweezers and the signal cycle using the DNAzyme tail were confirmed. Figure 2C demonstrates that the presence of all four strands of the DNA tweezers leads to a notable decrease in fluorescence signal, indicating that chains 2 and 4 are immobilized by chain 3. When any of the target *mecA*, H1 probe, H2 probe, or H3 probe were not present, the fluorescence signal remained consistently low. Upon the presence of all essential factors, the fluorescence signal exhibited a substantial increase, thereby activating the DNA tweezers.

Ultimately, the practicality of this approach for detecting *mecA* in SA in their natural environment was confirmed. The findings demonstrated that the introduction of the *mecA* detection-related probe into SA resulted in a notable and progressively intensified fluorescence signal on the plate, which ultimately reached its highest point after 60 min (Fig. 2D).

### The Optimization of the Biosensor Parameters

**Fig. 3:**
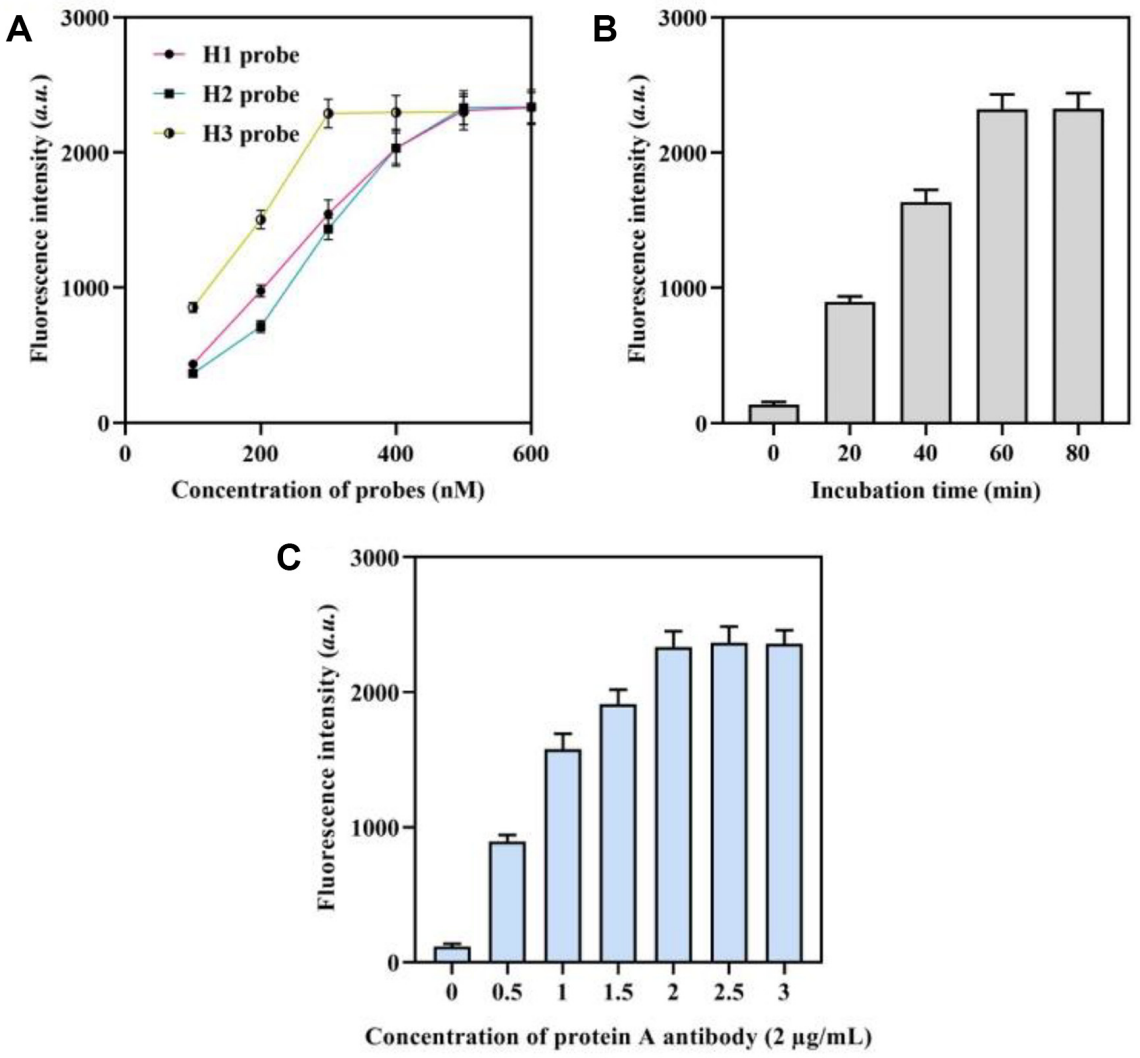
Optimization of experimental parameters. Fluorescence intensities of the approach for *mecA* gene detection with different concentrations of probes (**A**) with different incubation time (**B**) and with different concentrations of protein A antibody concentrations (**C**). “0” was the fluorescence intensity when target was absent in the sensing system (background signal).

In order to enhance the stability and optical distinction of the spectra, modifications were made to the biosensor’s reaction conditions. Additionally, the performance of the biosensor was examined in relation to various parameters, such as the concentrations of H probes (H1, H2, and H3 probes), and the concentration of protein A antibody. Fig. 3A demonstrates that the fluorescence intensity increases as the concentration of the H1 probe is increased. The fluorescence reaction achieved a state of dynamic equilibrium when the concentration of H1 probes exceeded 500 nM. Furthermore, the optimal concentrations of the H2 probe and H3 probe were found to be 500 nM and 300 nM, respectively. It is important to mention that as the concentration of the H3 probe approaches 300 nM, the fluorescence signal steadily rises. However, the background signal also increases simultaneously. Thus, a concentration of 300 nM was selected as the most suitable for the H3 probe. To enhance the efficiency of the *mecA* detection method, we assessed the fluorescence intensity at various time intervals. Fig. 3B demonstrates that the fluorescence intensity reached its highest point after 60 min, which was chosen for subsequent studies. The optimal concentration of the protein A antibody was discovered to be 2 μg/ml (Fig. 3C).

### In Vitro Analytical Performance

**Fig. 4:**
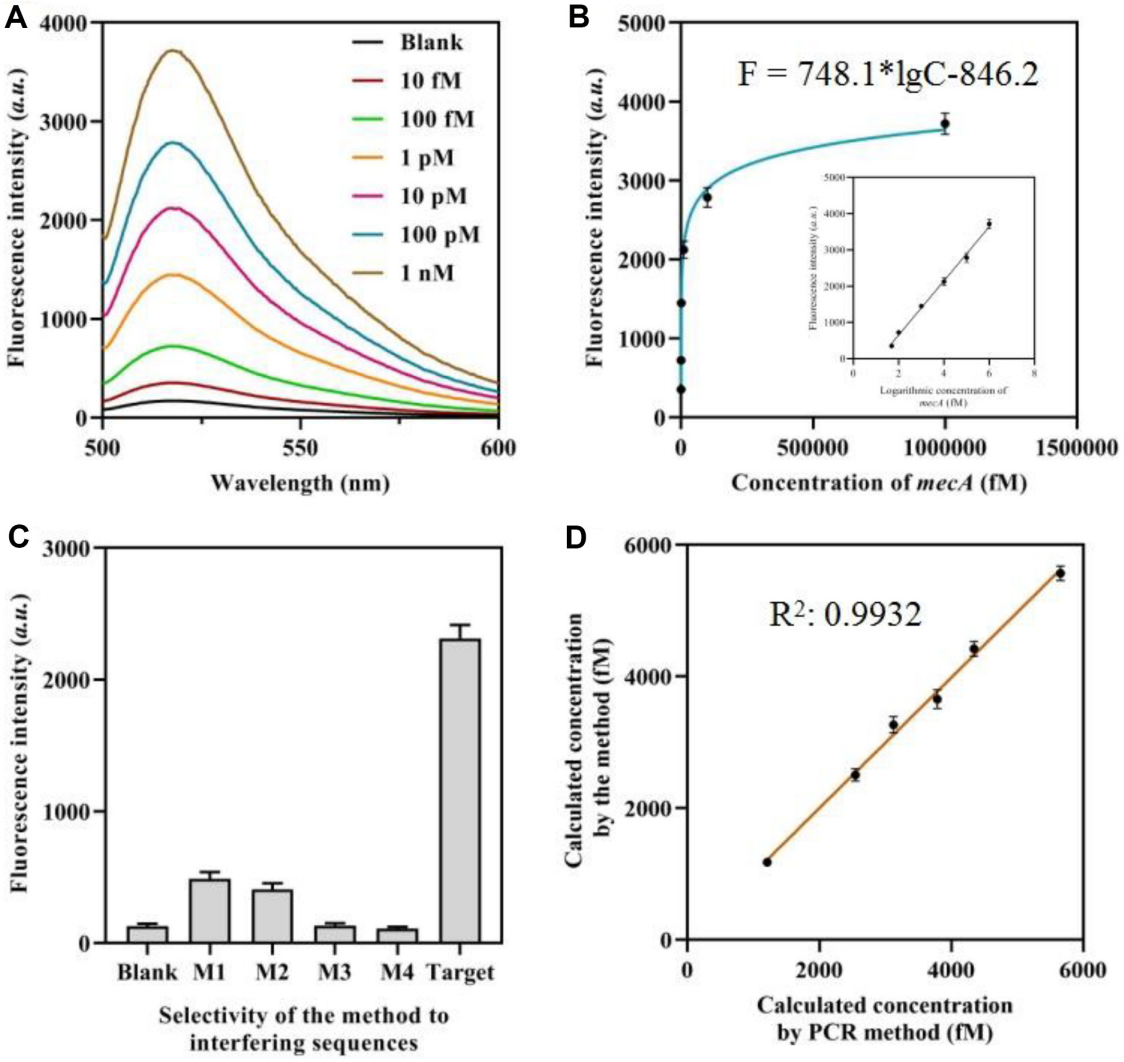
Analytical performance of the approach. (**A**) Fluorescence spectrum of the approach when detecting different concentrations of *mecA*. (**B**) Correlation equation between the fluorescence intensities and concentration of *mecA*. (**C**) Fluorescence intensities of the approach when detecting different *mecA*. (**D**) Correlation between the calculated *mecA* by the method and by PCR method.

The analytical performance of the proposed biosensor for detecting *mecA* was investigated under the optimal experimental conditions. Fig. 4A demonstrates a consistent increase in fluorescence intensity as the concentration of *mecA* varied from 10 fM to 1 nM. Fig. 4B demonstrates a strong linear correlation between the fluorescence intensity and the logarithm of *mecA* concentration. The regression equation is expressed as F = 748.1*lgC-846.2 (R^2^ = 0.9948). In this equation, F represents the fluorescence intensity value, while C represents the logarithm of the *mecA* concentration. The calculated limit of detection (LOD) for our new technology is 1.5 fM, making it one of the most sensitive *mecA* sensing systems in comparison to numerous other techniques.

The selectivity of the sensor described in this study was examined by assessing similar *mecA* sequences that have 1 to 4 base pairs mismatched with *mecA* gene (M1, M2, M3, M4) at equal concentrations. The fluorescence intensity obtained is shown in Fig. 4C. The test sample containing the target *mecA* gene exhibited the most pronounced fluorescence signal. The signals produced by interfering genes accounted for roughly 18%, 21%, 6%, and 8% of the signals generated by the target *mecA*, respectively.

In order to validate the performance and feasibility of the biosensor that was developed to detect *mecA*, a series of diluted (1%) human serum samples containing *mecA* were analyzed. The high degree of consistency between the calculated *mecA* concentration using the proposed method and that determined by the PCR method is illustrated in Figure 4D. This indicates that the new method possesses considerable practical value. Significantly, the results demonstrated that the sensing system retained its capacity for precise molecular recognition of the target gene, even when exposed to authentic human serum.

### The *mecA* Analysis on Plate

**Fig. 5:**
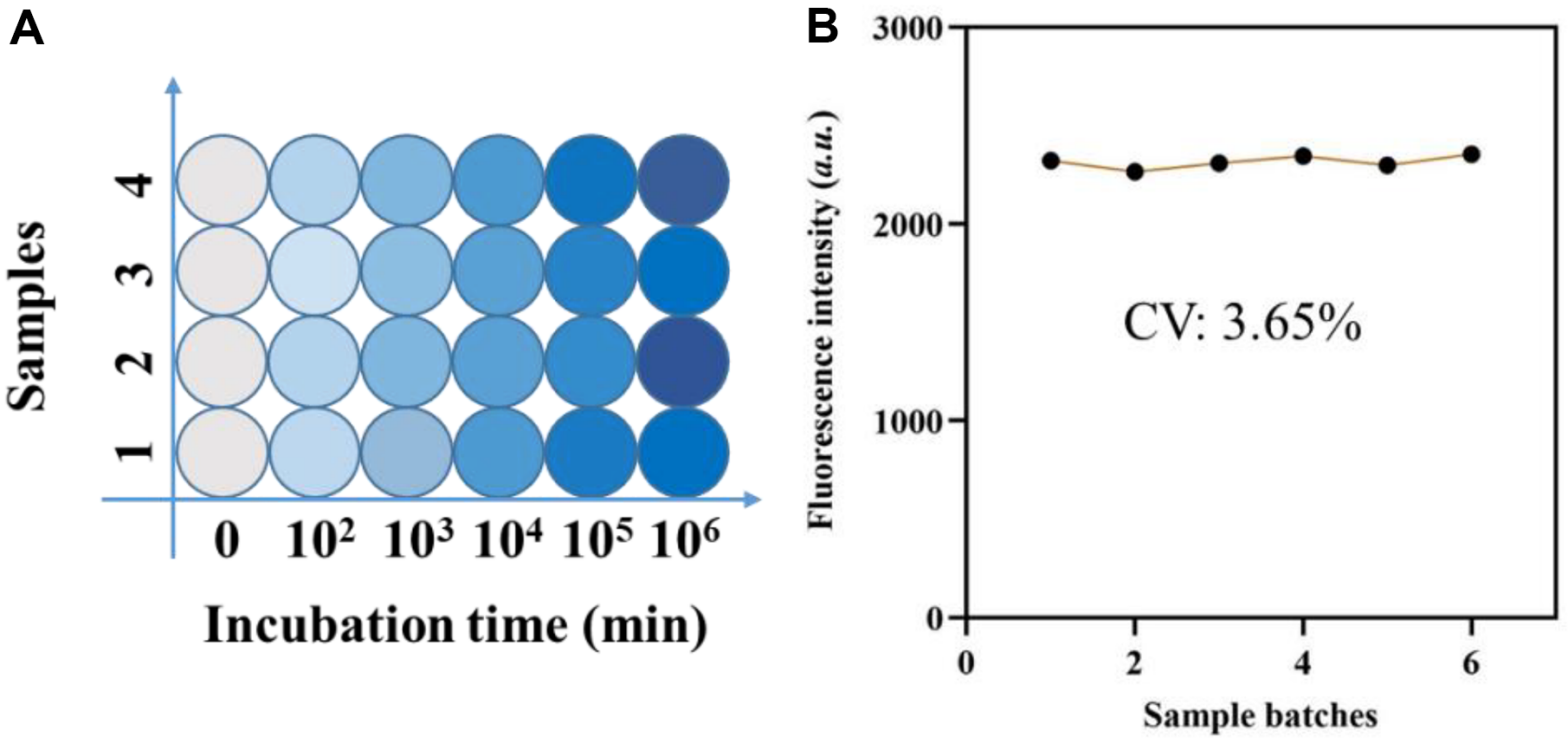
*In situ* *mecA* detection. (**A**) Fluorescence of the approach when detecting different concentrations of *mecA*. (**B**) Fluorescence intensities of the approach when detecting 6 samples batches.

The expression level of *mecA* in SA can be determined by recording the fluorescence signal on the plate surface. To simulate the concentration of *mecA*, we placed varying amounts of SA onto the plate surface and assumed that *mecA* concentrations in each SA separated from the same batch were normally distributed. As shown in Fig. 5A, the fluorescence signals obtained on plates loaded with varying concentrations of SA showed concentration-dependent variations, indicating that the fluorescence intensity rose as the concentration of SA *mecA* increased. We tested the repeatability of the procedure by measuring *mecA* quantities in ten samples. The results revealed that the approach maintained a high repeatability (coefficient of variation: 3.65%), emphasizing its usefulness in clinical settings (Fig. 5B).

## Conclusion

In brief, the integration of triple-branch CHA into DNAzyme switch controllable DNA tweezers led to the development of a fluorescent biosensor designed for the detection of *mecA*. The biosensor operates by immobilizing SA from samples onto the plate surface via the protein A antibody. By integrating the specific target recognition-initiated triple-branch CHA and DNA tweezer-based signal recycling, the method enables enzyme-free, highly sensitive detection of *mecA*. When detecting *mecA*, the proposed biosensor demonstrated notable benefits compared with traditional DNAzyme-based method, including enhanced sensitivity, exceptional selectivity, a straightforward procedure, and time efficiency (Table S2). Furthermore, the practicality of the biosensor was further demonstrated through its implementation to the cereal samples. Furthermore, this approach showcased the functionality and construction of a DNA tweezers and held promise for the early analysis of methicillin-resistance of SA during the nursing of pneumonia.

## Supplemental Materials

Supplementary data for this paper are available on-line only at http://jmb.or.kr.



## References

[1] Ahmad-Mansour N, Loubet P, Pouget C, Dunyach-Remy C, Sotto A, Lavigne JP (2021). *Staphylococcus aureus* toxins: an update on their pathogenic properties and potential treatments. Toxins (Basel).

[2] Chalmers SJ, Wylam ME (2020). Methicillin-resistant *Staphylococcus aureus* infection and treatment options. Methods Mol. Biol..

[3] Parker D, Prince A (2012). Immunopathogenesis of *Staphylococcus aureus* pulmonary infection. Semin. Immunopathol..

[4] Bassetti M, Labate L, Melchio M, Robba C, Battaglini D, Ball L (2022). Current pharmacotherapy for methicillin-resistant *Staphylococcus aureus* (MRSA) pneumonia. Expert Opin. Pharmacother..

[5] Dangerfield B, Chung A, Webb B, Seville MT (2014). Predictive value of methicillin-resistant *Staphylococcus aureus* (MRSA) nasal swab PCR assay for MRSA pneumonia. Antimicrob. Agents Chemother..

[6] He H, Wunderink RG (2020). *Staphylococcus aureus* pneumonia in the community. Semin. Respir. Crit. Care Med..

[7] Guo Y, Song G, Sun M, Wang J, Wang Y (2020). Prevalence and therapies of antibiotic-resistance in *Staphylococcus aureus*. Front. Cell. Infect. Microbiol..

[8] Lee AS, de Lencastre H, Garau J, Kluytmans J, Malhotra-Kumar S, Peschel A (2018). Methicillin-resistant *Staphylococcus aureus*. Nat. Rev. Dis. Primers.

[9] Peacock SJ, Paterson GK (2015). Mechanisms of methicillin resistance in *Staphylococcus aureus*. Annu. Rev. Biochem..

[10] Choe H, Kobayashi N, Ito Y, Ike H, Tezuka T, Takeyama M (2022). Detection of *mecA* and 16S rRNA genes using real-time PCR can be useful in diagnosing iliopsoas abscess, especially in culture-negative cases: RT-PCR for iliopsoas abscess. Biomed Res. Int..

[11] Fueller J, Herbst K, Meurer M, Gubicza K, Kurtulmus B, Knopf JD (2020). CRISPR-Cas12a-assisted PCR tagging of mammalian genes. J. Cell Biol..

[12] Han HW, Chang HC, Chang TC (2016). Identification of *Staphylococcus* spp. and detection of *mecA* by an oligonucleotide array. Diagn. Microbiol. Infect. Dis..

[13] Monecke S, Muller E, Schwarz S, Hotzel H, Ehricht R (2012). Rapid microarray-based identification of different *mecA* alleles in Staphylococci. Antimicrob. Agents Chemother..

[14] Jet T, Gines G, Rondelez Y, Taly V (2021). Advances in multiplexed techniques for the detection and quantification of microRNAs. Chem. Soc. Rev..

[15] Li Q, Zhou D, Pan J, Liu Z, Chen J (2018). Ultrasensitive and simple fluorescence biosensor for detection of the *mecA* gene of *Staphylococcus aureus* by using an exonuclease III-assisted cascade signal amplification strategy. Analyst.

[16] Lin Q, Xu P, Li J, Chen Y, Feng J (2017). Direct bacterial loop-mediated isothermal amplification detection on the pathogenic features of the nosocomial pathogen - Methicillin resistant *Staphylococcus aureus* strains with respiratory origins. Microb. Pathog..

[17] Pan J, Bao D, Bao E, Chen J (2021). A hairpin probe-mediated DNA circuit for the detection of the *mecA* gene of *Staphylococcus aureus* based on exonuclease III and DNAzyme-mediated signal amplification. Analyst.

[18] Weng X, Lou J, Zhang J, Wang Y, Wu Q (2023). Sensitive and portable detection of bacteria using exonuclease-III (Exo-III) assisted signal amplification and personal glucose meters. Mol. Biotechnol..

[19] Chen H, Sun X, Cai R, Tian Y, Zhou N (2019). Switchable DNA tweezer and G-quadruplex nanostructures for ultrasensitive voltammetric determination of the K-ras gene fragment. Mikrochim. Acta.

[20] Chen R, Mao Z, Lu R, Wang Z, Hou Y, Zhu W (2022). Simple and programmed three-dimensional DNA tweezer for simultaneous one-step detection of ochratoxin A and zearalenone. Spectrochim. Acta A Mol. Biomol. Spectrosc..

[21] Li J, Wang W, Zhang H, Lu Z, Wu W, Shu M (2020). Programmable DNA tweezer-actuated SERS probe for the sensitive Detection of AFB(1). Anal. Chem..

[22] Yao Y, Liu Y, Liu X, Zhang X, Shi P, Zhang X (2024). Bubble DNA tweezer: a triple-conformation sensor responsive to concentration-ratios. iScience.

