# Supplementary material for: Triple-Branch Catalytic Assembly DNAzyme Motivated DNA Tweezer for Sensitive and Reliable mecA Gene Detection in Staphylococcus aureus
Source: J Microbiol Biotechnol. 2024 Oct 1;34(12):2450–6. doi: 10.4014/jmb.2409.09008 (PMC11729337; doi:10.4014/jmb.2409.09008)
Supplement: Supplementary file 1 [file jmb-34-12-2450-supple.pdf]

J. Microbiol. Biotechnol. 2024. 34(12): 2450–2456
https://doi.org/10.4014/jmb.2409.09008

Supplementary Tables

Table S1. Sequences of nucleic acid molecules involved in the text.

Title	Sequences (5' to 3')
H1	CTC TAT CAT TAT CTT GCT TCA TCT TCA TCA AGA TAA TGA TAG AGA CCG ACA CTC
H2	GCT TCA TCT TCA TCT TCT CTA TCA TTA TCT TGA TGA AGA TGA AGC AAG ATA AT
H3	GAG TGT CGG AGA TGA AGA TGA AGC CAT CGT CGG TAG CCT GT GCT TCA TCT TCA TGT
Target gene (*mecA*)	GAG TGT CGG TCT CTA TCA TTA TCT T
1 sequence in DNA Tweezer	GTTGGAGCGACATTAGAGA-GCTACAA-FAM
2 sequence in DNA Tweezer	DABCYL-GTAGCCT-CCTGTCCTATCTATGATGG
4 sequence in DNA Tweezer	CTAATGTCGCTCCAAC-AA-CCATCATAGATAGGAC
3 sequence in DNA Tweezer	TT-GTAGC-ACAG-GCTAC-CG
M1	GAC TGT CGG TCT CTA TCA TTA TCT T
M2	GAC AGT CGG TCT CTA TCA TTA TCT T
M3	GAC AGT CCG TCT CTA TCA TTA TCT T
M4	GAC AGT CCC TCT CTA TCA TTA TCT T

Table S2. A brief comparisons of the method with former ones.

Title	Mechanism	Sensitivity	Signals	Enzymes	Advantages and disadvantages	Ref
The method	Triple-branch CHA+ DNA tweezer	1.5 fM	Fluorescence signal	Enzyme-free	Advantages: high sensitivity; enzyme-free; easy-to-design.<br>Disadvantages: could be further applied for point of care testing.	
A hairpin probe-mediated DNA circuit	Exonuclease III and DNAzyme-mediated signal amplification	0.5 fM	Fluorescence signal	Exonuclease III	Advantages: high sensitivity.<br>Disadvantages: require enzymes for signal amplification; low stability.	[1]
PGMs-based method	Exonuclease-III-assisted signal recycles+ CHA	4.36 fM	Fluorescence signal	Exonuclease III	Advantages: high sensitivity; portability.<br>Disadvantages: require enzymes for signal amplification; low stability; complicated procedures.	[2]
Microchip electrophoretic detection	Isothermal strand-displacement polymerase reaction	12.3 pM	Electrophoretic changes	KF	Advantages: portability.<br>Disadvantages: require enzymes for signal amplification; low stability; complicated procedures; low sensitivity.	[3]
Colorimetric approach	Exonuclease-III catalyzed signal cascade	3.4 fM	Color changes	Exonuclease III	Advantages: direct read results by naked eyes.<br>Disadvantages: require enzymes for signal amplification; low stability; complicated procedure.	[4]

CHA, catalytic hairpin assembly; PGMs, personal glucose meters

References

1. Pan J, Bao D, Bao E, Chen J. 2021. A hairpin probe-mediated DNA circuit for the detection of the mecA gene of *Staphylococcus aureus* based on exonuclease III and DNAzyme-mediated signal amplification. *Analyst.* **146:** 3673-3678.
2. Su J, Zheng W. 2023. Dual-Toehold-Probe-Mediated Exonuclease-III-Assisted Signal Recycles Integrated with CHA for Detection of mecA Gene Using a Personal Glucose Meter in Skin and Soft Tissue Infection. *J. Microbiol. Biotechnol.* **33:** 1692-1697.
3. Lu Y, Luo F, Li Z, Dai G, Chu Z, Zhang J*, et al.* 2021. Ultrasensitive microchip electrophoretic detection of the mecA gene in methicillin-resistant *Staphylococcus aureus* (MRSA) based on isothermal strand-displacement polymerase reaction. *Talanta* **222:** 121686.
4. Deng X, Yao X. 2024. A simple and sensitive colorimetric approach for mecA gene analysis via exonuclease-III catalyzed signal cascade. *Anal. Biochem.* **687:** 115453.
